# Management of a Muskellunge Fish Bite to the Thumb With Subsequent Flexor Tendon Laceration

**DOI:** 10.7759/cureus.75541

**Published:** 2024-12-11

**Authors:** Nicholas W Miller, Zachary A Koenig, Kerri M Woodberry

**Affiliations:** 1 Plastic Surgery, West Virginia University School of Medicine, Morgantown, USA

**Keywords:** fish bite, flexor tendon laceration, infection, muskellunge fish, thumb

## Abstract

Despite frequent occurrences, especially throughout the Appalachian region, fish bite injuries remain largely underreported. Muskellunge anglers are at a particularly heightened risk due to the fish’s large mouth and notably sharp teeth. We present a case of a male who sustained an injury to the right volar thumb following a muskellunge bite. Notably, the patient denied pain and tenderness following the incident, but had an inability to flex the interphalangeal joint. Following exploration, the patient underwent irrigation, debridement, and repair of a flexor pollicis longus tendon laceration. Subsequently, the patient required occupational therapy to improve range of motion, and antimicrobial therapy for surgical prophylaxis. Themedical and surgical management of each fish bite injury undoubtedly represents a unique clinical puzzle for healthcare professionals that is dependent upon the severity of the bite, regional anatomy involved, and time course in seeking treatment. Creating a record of notable identified cases in the scientific literature could be pertinent for treatment of patients with similar presentations.

## Introduction

Fishing is a significant commercial and recreational pursuit within Appalachian culture. Despite the revenue and enjoyment the activity provides, it is also associated with substantial safety concerns and a notable number of documented medical emergencies. Among the potential injuries, fish bites are relatively common [1]. For example, in a survey of 81 commercial fishermen in North Carolina, 69 reported experiencing a serious bite or sting associated with their work [2]. Another study analyzed 204 wounds caused by fish among professional fishermen and found that 97% occurred during handling while removing fishing gear. The same report noted that 60.3% of the injuries involved the hand, and 84% of individuals did not seek medical attention after the incident [3].

When addressing fish bites, prompt management of the direct trauma is crucial. Additionally, care must be taken to prevent or treat any secondary infections that may arise. These infections could result from organisms colonizing the attacking fish or those present in the surrounding aquatic environment [4]. Interestingly, there are reports of painless fish bites in humans, particularly by Meiacanthus spp., which release chemicals that interact with delta opioid receptors in their prey, inducing analgesia and hypotensive effects [5]. Furthermore, other studies have detected an abundance of opioids and their metabolites in waters, especially in areas severely affected by the opioid epidemic [6]. However, limited data exist on the specific quantities of these substances in the environment or their potential to bioaccumulate in fish, making it unclear whether they significantly impact human health.

This report highlights a unique case of a fish bite resulting in a complete rupture of a flexor tendon in the hand. While fish bite injuries are commonly superficial, this case underscores the potential for deeper soft tissue damage that may require surgical intervention. The primary purpose of this case presentation is to emphasize the importance of prompt recognition, timely surgical management, and targeted rehabilitation to optimize functional recovery. By sharing this case, we aim to provide insight into the complexities of managing similar injuries, particularly in regions where fishing is a prevalent activity.

## Case presentation

A 42-year-old right-handed male presented to an outside facility immediately following an injury that occurred while he was muskellunge fishing. He initially reported that he had caught the fish, and while maneuvering the removal of the hook, his hand slipped, and the fish tooth pierced his right volar thumb. His wound was washed out at bedside, and his metacarpophalangeal (MCP) and interphalangeal (IP) joints of the right thumb were noted to have limited range of motion. The patient was administered a tetanus toxoid/diphtheria toxoid/acellular pertussis vaccination, provided a prescription for two weeks of clindamycin and levofloxacin for broad-spectrum coverage against organisms commonly associated with animal bites, and discharged home.

Four days later, the patient presented to our facility with a persistent limited range of motion of the right thumb IP joint. His range of motion of the MCP had improved. A 1-centimeter superficial laceration was identified over the volar surface of the right thumb. Sensation was intact, and capillary refill was normal (2 seconds or less) bilaterally. There were no foreign bodies or osseous abnormalities visible on X-ray of the right thumb. The patient had a past medical history of hypertension that was treated with lisinopril. He was a former user of smokeless tobacco. 

**Figure 1:**
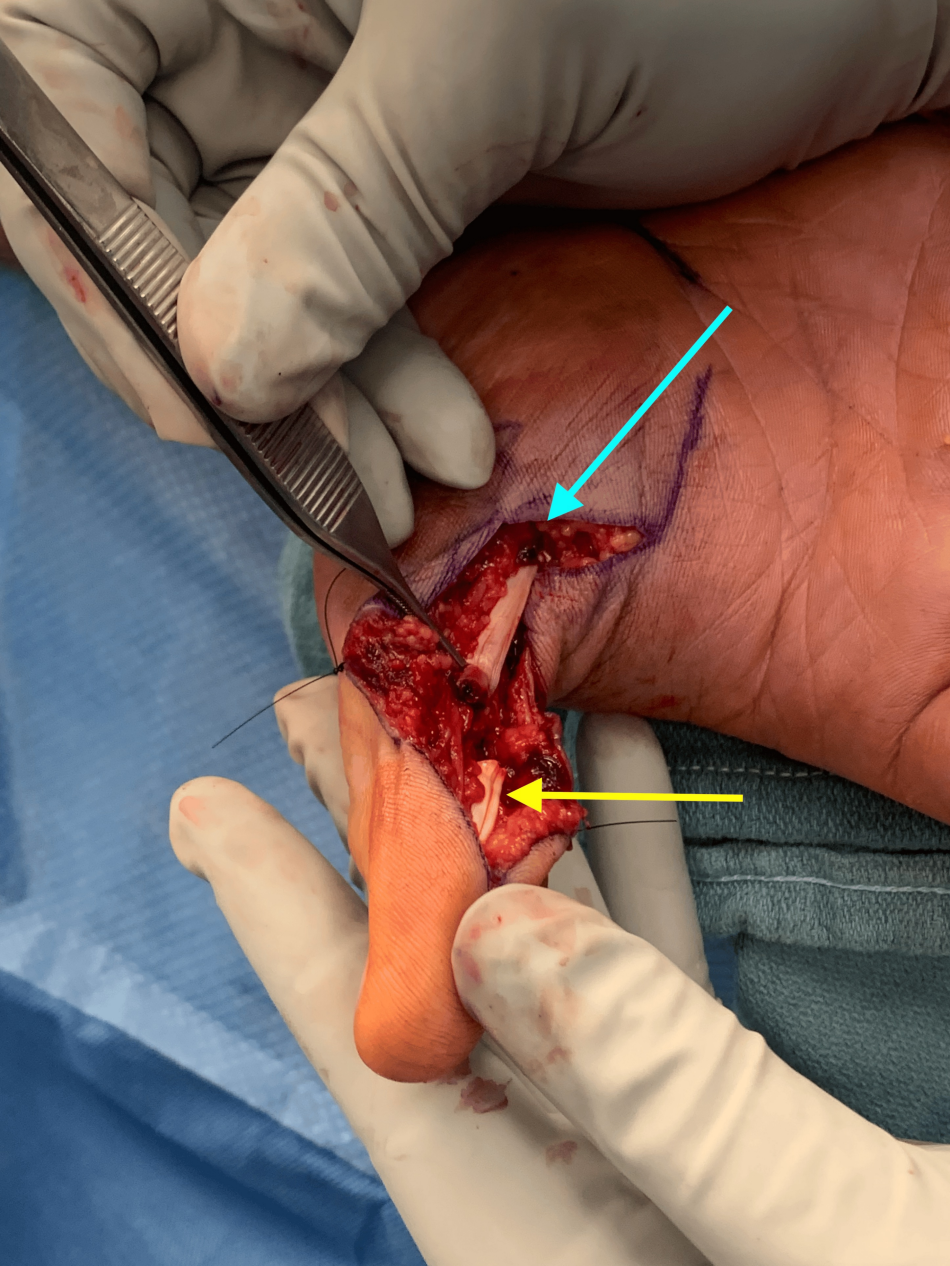
Intra-operative view of right hand. The flexor pollicis longus (FPL) tendon was found to be 100% lacerated just proximal to the right thumb interphalangeal (IP) joint. The blue arrow highlights the proximal end of the tendon, and the yellow arrow highlights the distal end of the tendon.

**Figure 2:**
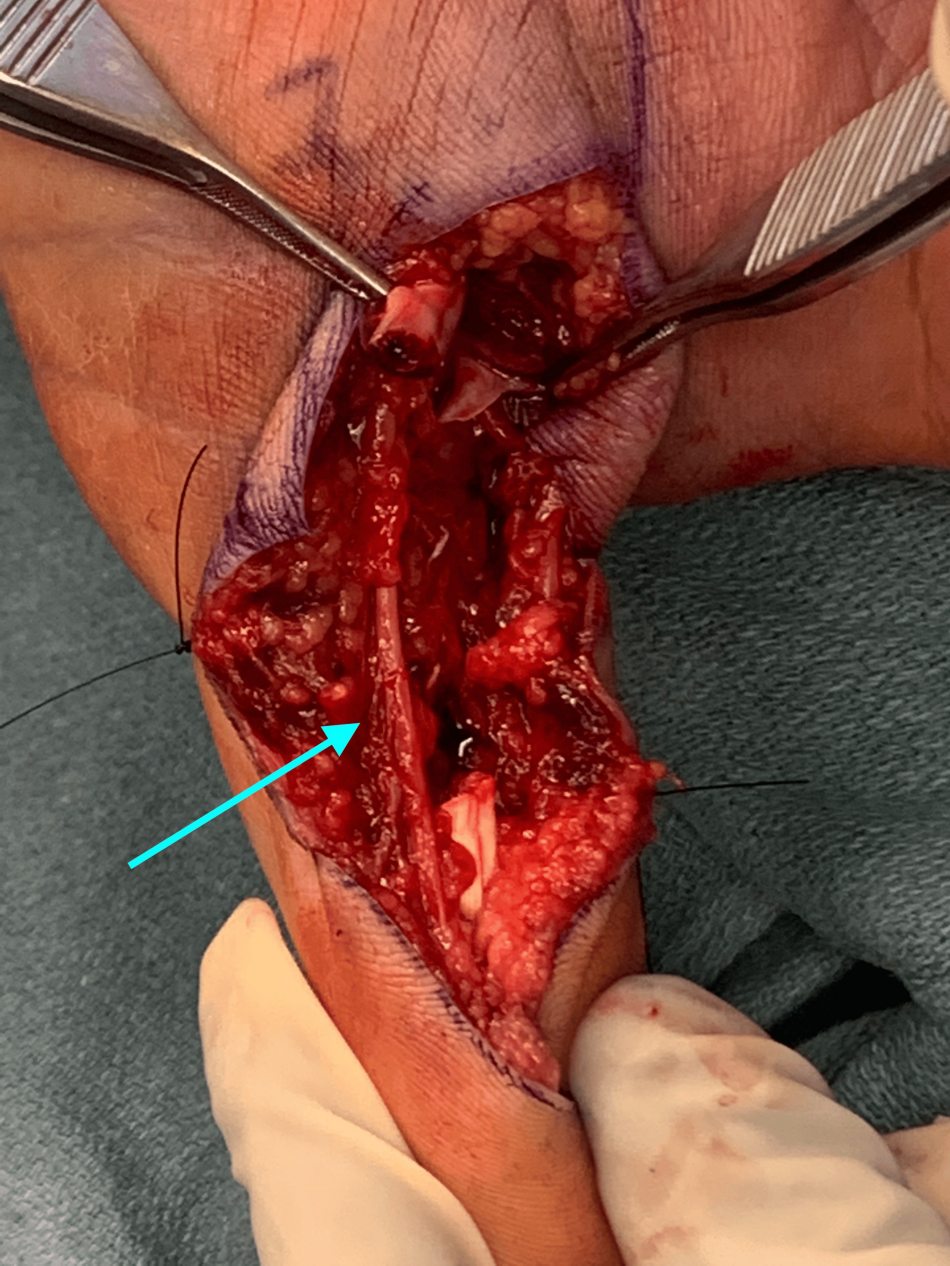
Intra-operative view of the right hand. The neurovascular island beside the lacerated flexor pollicis longus (FPL) was found intact without the need for repair. The blue arrow highlights the intact neurovascular island.

Suspecting possible laceration of the flexor pollicis longus (FPL) tendon, informed consent was obtained and a surgical exploration, irrigation, and debridement was performed the following day. Notable intraoperative findings included a hematoma, necrotic tissue, fluid with a thick viscous consistency, and a 100% lacerated FPL tendon just proximal to the IP joint (Figure 1). The neurovascular structures were intact (Figure 2). Specimens were collected for Gram staining, as well as aerobic, anaerobic, and acid-fast bacilli cultures. Immediate Gram stain results, available within 60 minutes of specimen collection, did not reveal any organisms but noted the presence of polymorphonuclear cells. The lacerated FPL tendon was repaired using a 3-0 Prolene locking modified Kessler suture and oversewn with a 6-0 Prolene epitendinous suture. The wound was irrigated with cefazolin to reduce bacterial load and provide prophylactic coverage against Gram-positive skin flora and then closed. The patient was placed in a dorsal blocking splint with the thumb in flexed position. Postoperatively, doxycycline was added to his existing regimen of clindamycin and levofloxacin to provide coverage for potential aquatic pathogens, including Vibrio, Mycobacterium marinum, and Aeromonas species, for a duration of 14 days after his operation.

Six days after the surgery, the patient returned to clinic and denied any significant complaints regarding pain or limited function. On examination, his laceration remained well approximated, sutures were intact, and his thumb was well perfused. However, at this visit, he noted a new onset of mild hyperesthesia over the distal aspect of the right thumb, which had not been present during the initial postoperative assessment. To alleviate the hyperesthesia, he was provided with instructions for a desensitization process, which involved repetitive sensory stimulation techniques to reduce hypersensitivity in the affected area. He was also advised to begin occupational therapy (OT) in two weeks while continuing to maintain the dorsal blocking splint.

Nineteen days later (post-operative day 39), the patient began OT. At this initial appointment, he reported being able to complete his activities of daily living at 50% of normal level; his Disabilities of the Arm, Shoulder, and Hand (DASH) score was 68.1 (range: 0-100 with higher scores indicating greater disability). Total passive motion (combination of flexion of MCP and IP) of the right thumb was measured to be 88 degrees without any active range of motion. The patient completed eight total sessions of OT over about four weeks.

At the final OT appointment, the patient reported being able to use his right hand for all activities and was satisfied with his progress. His DASH score was 6.8, total passive motion measured 130 degrees, and total active motion measured 99 degrees. A significant reduction in edema of the thumb was reported over the course of the patient’s care with OT. All microbial cultures obtained from the hand during the surgical exploration and debridement remained negative.

## Discussion

Rural West Virginia provides a unique challenge for healthcare professionals due to the wide array of pathology that presents in its underserved regions. Cases range from chronic diseases often exacerbated by limited healthcare access to unique injuries related to the region's natural environment and industries, such as fishing injuries, mining accidents, and agricultural trauma [7-10]. These diverse pathologies demand a broad and adaptable scope of practice for clinicians to manage complex cases with limited resources effectively. Studies have highlighted the challenges and variability in care provision in rural areas, emphasizing the need for a tailored approach to address the specific health disparities and environmental factors impacting these communities [1,11,12].

The muskellunge, *Esox masquinongy*, is a large predatory freshwater fish native to North America. Documented to grow to 30 kilograms and 140 centimeters in length, it is a strong fighter that is extremely difficult to catch [13]. It is possible that cases of bites to humans may be the fish mistaking people for prey. Notably, the muskellunge has a large mouth with characteristic teeth that have been described as “needle-like,” resembling those of canines [14]. These sharp teeth have the potential to easily cut human skin and underlying structures if fingers are trapped in their mouths during handling.

When assessing a patient bitten by a fish, it is important to consider the potential pathogens that may have been transmitted by the bite. Notably, the following bacteria have been described as being associated with freshwater fish and surrounding waters: *Vibrio vulnificus*, *Aeromonas hydrophila*, *Pseudomonas aeruginosa*, *Erysipelothrix rhusiopathiae*, *Edwardsiella tarda*, and *Mycobacterium marinum* [2,6]. To effectively prevent or treat the potential infections that may be acquired in a muskellunge bite, antibiotic coverage should empirically cover all potential organisms. *M. marinum* remains particularly difficult to culture clinically because it requires a special Löwesntein-Jensen medium and incubation at 28-30 °C for several weeks [15]. Treatment choices depend on the severity of infection. Historically, lesions that arise from *M. marinum* infection can be divided into three categories: type I lesions (self-limited verrucal), type II lesions (subcutaneous granuloma with or without ulceration), or type III lesions (deep involving tenosynovium, bursa, bones, or joints). Notably, the organism is resistant to traditional antituberculous medications, including isoniazid, streptomycin, and para-aminosalicylic acid [16].

Early recommendations for treatment included doxycycline, cotrimoxazole, or ciprofloxacin for superficial infection or rifampicin and ethambutol for deeper infections; however, failures of all of these regimens have been described [17]. Newer suggestions consider a combination of ciprofloxacin and clarithromycin to be most effective [18]. This combination would provide broad spectrum coverage of Gram-positive and Gram-negative organisms, including *Pseudomonas* and *Aeromonas* species, making it a good choice for empirical treatment following a fish bite. Treatment should typically continue for four to six weeks or even up to 18 months for deeper infections [17,18]. Following surgery, doxycycline was added to the existing regimen of clindamycin and levofloxacin. This combined regimen was continued for a total of two weeks post-operatively. None of the microbial cultures obtained from the hand during surgical exploration were positive for any organisms.

In addition to the aforementioned infections, it is also important to assess the need for tetanus prophylaxis. While the occurrence of tetanus after bites from nonvenomous animals is rare, wounds prone to infection by *Clostridium tetani* include those that persist for at least six hours after initial injury, are greater than 1 centimeter in depth, have been contaminated, display necrotic tissue, and show signs of infection [19]. These chronic wounds that result in necrosis can create an anaerobic environment that would allow *C. tetani* to colonize and thrive [4]. The patient’s initial treatment at the outside facility included a tetanus diphtheria and acellular pertussis vaccination to aid in preventing disease.

## Conclusions

This case report highlights the presentation, treatment, and follow-up of a patient with a rare fish bite injury complicated by a flexor pollicis longus rupture. The interplay between environmental, anatomical, and microbial factors underscores the complexity of managing fish bite injuries in both acute and long-term settings. As fishing grows in popularity as a recreational activity in Appalachia and beyond, clinicians are likely to encounter similar cases, emphasizing the importance of standardized treatment guidelines. Documenting these injuries enhances awareness of the risks associated with fishing and informs optimal management strategies. This case reinforces the need for a multidisciplinary approach, including surgical repair, antimicrobial coverage, and rehabilitation, to achieve full functional recovery.

## References

[1] Koenig ZA, Henderson JT, Meaike JD, Gelman JJ (2024). Challenges in rural plastic surgery: availability, scope of practice, and motivating factors. Curr Probl Surg.

[2] Burke WA, Griffith DC, Scott CM, Howell ER (2006). Skin problems related to the occupation of commercial fishing in North Carolina. N C Med J.

[3] Costa TN, Jacó TR, Casas AL, Bernarde PS (2019). Injuries caused by fish to fishermen in the Vale do Alto Juruá, Western Brazilian Amazon. Rev Soc Bras Med Trop.

[4] Domingos MO, Franzolin MR, dos Anjos MT (2011). The influence of environmental bacteria in freshwater stingray wound-healing. Toxicon.

[5] Casewell NR, Visser JC, Baumann K (2017). The evolution of fangs, venom, and mimicry systems in blenny fishes. Curr Biol.

[6] Simpson J, Simpson BS, Gerber C (2024). Effect of secondary and tertiary wastewater treatment methods on opioids and the subsequent environmental impact of effluent and biosolids. Chemosphere.

[7] Koenig ZA, Lokant BT, Weaver S, Brooke SM, Uygur HS (2024). Surgical guide splint fabrication via virtual surgical planning for complex mandible fractures in the trauma setting. J Craniofac Surg.

[8] Koenig ZA, Robertson GA, Koenig NI, Durkin PR, McCarthy R (2021). Massive gasoline ingestion in a 64-year-old female: an explosive situation. Cureus.

[9] Koenig ZA, Burns JC, Hayes JD (2022). Necrotic granulomatous inflammation after use of small intestine submucosa matrix for recurrent compression neuropathy. Plast Reconstr Surg Glob Open.

[10] Shabih S, Lewis B, Koenig ZA, Brooke SM, Meltzer H, Uygur HS (2024). Pott puffy tumor secondary to cranial vault reconstruction in a patient with metopic craniosynostosis. J Craniofac Surg.

[11] Van Antwerp E, Koenig ZA, McCarthy R (2021). Modern medical miracle: matched unrelated donor hematopoietic stem cell transplant after aplastic anemia. Cureus.

[12] Salisbury FK, Koenig ZA, Uygur HS (2024). Management of frontal bone exposure following paramedian forehead flap for nasal reconstruction. J Craniofac Surg.

[13] New JG, Alborg Fewkes L, Khan AN (2001). Strike feeding behavior in the muskellunge, Esox masquinongy: contributions of the lateral line and visual sensory systems. J Exp Biol.

[14] Vinson MR, Angradi TR (2014). Muskie lunacy: does the lunar cycle influence angler catch of muskellunge (Esox masquinongy)?. PLoS One.

[15] Simpson PA, Przybylo M, Blanchard TJ, Wingfield T (2017). The brief case: a fishy tale prevents digital doom following Polly’s Peck-the importance of pets in a comprehensive medical history. J Clin Microbiol.

[16] Hurst LC, Amadio PC, Badalamente MA, Ellstein JL, Dattwyler RJ (1987). Mycobacterium marinum infections of the hand. J Hand Surg Am.

[17] Kumar V, Taranu R (2005). Masquerading Mycobacterium: plastic surgeon to the rescue. Can J Plast Surg.

[18] Laing RB, Flegg PJ, Watt B, Leen CL (1997). Antimicrobial treatment of fish tank granuloma. J Hand Surg Br.

[19] Collins AN, Rose WD (2004). Toxic shock syndrome in an adult male secondary to puncture wound. W V Med J.

